# Copyright

**Published:** 1867

**Authors:** 


*Copyright.*—The translation of *Desormeaux Lessons on the Endoscope* has been copyrighted for publication in book form as soon as completed in the JOURNAL. The notice of entry was accidentally omitted in this number of the JOURNAL. As the whole treatise would occupy rather too much space for the ordinary compass of the JOURNAL, a considerable portion will be added as a supplement to one or more numbers, without additional charge upon our subscribers. Aside from the illustrations of the *Endoscope*, it may be safely stated that these lectures contain the best analysis of urethral affections, with references also to analogous changes in other mucous surfaces, which has yet been given to the profession.

